# Paroxysmal slow wave events are associated with cognitive impairment in patients with obstructive sleep apnea

**DOI:** 10.1186/s13195-022-01153-x

**Published:** 2022-12-30

**Authors:** Mengfan Li, Zhuoran Sun, Hairong Sun, Guochen Zhao, Bing Leng, Tengqun Shen, Song Xue, Huimin Hou, Zhenguang Li, Jinbiao Zhang

**Affiliations:** 1 Department of Neurology, Weihai Municipal Hospital, Cheeloo College of Medicine, Shandong University, Weihai, 264200 Shandong China; 2 School of Ocean Engineering, Harbin Institute of Technology at Weihai, Weihai, 264209 Shandong China; 3 Department of Resident Standardized Training Management, Weihai Municipal Hospital, Cheeloo College of Medicine, Shandong University, Weihai, 264200 Shandong China; 4 Weifang Medical University, Weifang, 261053 Shandong China; 5 Department of Radiology, Weihai Municipal Hospital, Cheeloo College of Medicine, Shandong University, Weihai, 264200 Shandong China

**Keywords:** Obstructive sleep apnea, Blood-brain barrier, Cognitive impairment, Paroxysmal slow wave event

## Abstract

**Background:**

Increasing evidence has supported a link between obstructive sleep apnea (OSA) and cognition, and blood-brain barrier (BBB) dysfunction which can be reflected by paroxysmal slow wave events (PSWEs) may be a potential mechanism. The purpose of our study was to investigate the correlation between the PSWEs and cognitive impairment in patients with OSA, with a focus on the possible mechanism.

**Methods:**

In total, 339 subjects with subjective snoring complaints from the Sleep Medicine Center underwent magnetic resonance imaging and whole-night polysomnography. OSA was defined as apnea-hypopnea index (AHI) ≥ 5 events/h. MCI was defined as the MoCA < 26 and met the criteria: (1) subjective cognitive impairment; (2) objective impairment in one or more cognitive domains; (3) slightly impaired complex instrumental daily abilities, but independent daily living abilities; and (4) no dementia. The PSWEs calculated by self-developed Python scripts were defined for EEG recordings as a median power frequency of < 6 Hz for more than five consecutive seconds. Serum cyclophilin A (CyPA) and matrix metalloproteinase-9 (MMP-9) levels and amyloid-β 42 levels in neuron-derived exosomes were determined. The participants who received continuous positive airway pressure (CPAP) were followed up and their PSWEs were recalculated after 1 year of treatment.

**Results:**

A total of 339 participants were divided into the OSA+MCI group (*n* = 157), OSA-MCI group (*n* = 118), and controls (normal cognitive state without OSA) (*n* = 64). The total PSWEs and the occurrence per minute of PSWEs at stage REM in the OSA+MCI group were higher than those in the OSA-MCI and control groups. The duration ratio of PSWEs at stage REM in the OSA+MCI group significantly increased. The total PSWEs and PSWEs at the F4-M1, O1-M2, and O2-M1 channels in stage REM were independently associated with cognitive impairment in OSA patients. There were positive correlations between the PSWEs and serum CyPA and MMP-9 levels in patients with OSA. The mediation analysis showed that the relationship between mean SaO_2_ and percentage of sleep time spent with oxygen saturation <90% with MoCA scores was mediated by the total PSWEs (proportion of mediation 77.89% and 82.89%). The PSWEs were negatively correlated with global cognitive performance and cognitive subdomains. After 1 year of CPAP treatment, the total PSWEs, PSWEs in stage REM, and serum CyPA and MMP-9 levels decreased significantly, and MoCA scores were improved compared with baseline.

**Conclusions:**

The PSWEs were implicated in cognitive impairment in patients with OSA, and the mechanisms of cognitive impairment due to hypoxia in OSA patients could be BBB dysfunction. The PSWEs can be used as a marker of cognitive impairment in patients with OSA.

**Trial registration:**

This trial is registered on the Chinese Clinical Trial Registry, number ChiCTR1900021544. The trial was registered on February 27, 2019.

## Statement of significance

An increasing number of studies support the link between obstructive sleep apnea (OSA) and cognitive impairment, and the underlying mechanisms may involve blood-brain barrier dysfunction. This study analyzed EEG spectral features during a night of sleep in order to identify the correlation between paroxysmal slow wave events (PSWEs) and cognitive impairment in patients with OSA. Our study shows associations of increased PSWE occurrences with cognitive impairment in patients with OSA, and the PSWEs was correlated with levels of cognitive impairment. The mechanisms of cognitive impairment due to hypoxia in OSA patients could be blood-brain barrier dysfunction reflected by the PSWEs. The results suggest that the PSWEs may be a marker for cognitive impairment in OSA patients in assessing disease burden to guide therapeutic decisions.

## Introduction

Obstructive sleep apnea (OSA), a highly prevalent worldwide public health problem, is characterized by repetitive episodes of apnea-hypopnea due to partial or complete upper airway obstruction during sleep, resulting in recurrent intermittent hypoxia and sleep fragmentation [1]. Research on OSA is very important because its incidence and prevalence increase gradually, and it is associated with many chronic diseases and cognitive impairment, particularly in attention, episodic memory, and executive function [2]. Indeed, a great deal of research has supported an increased risk of developing mild cognitive impairment (MCI) and dementia in OSA patients compared with non-OSA individuals [3, 4], and the risk of cognitive impairment in OSA patients can increase by 30% [2].

The blood-brain barrier (BBB) is a selective diffusion barrier located in the brain capillaries, and it maintains homeostasis by tightly regulating the movement of molecules, ions, and cells between the blood and the central nervous system [5, 6]. Thus, BBB dysfunction can have adverse effects on central nervous system function. A recent study proposed that BBB breakdown is an early biomarker of human cognitive dysfunction independent of amyloid-β (Aβ) and tau, which are classic Alzheimer’s disease (AD) biomarkers [7]. BBB dysfunction has been well documented in OSA patients [8, 9]. It is generally believed that intermittent hypoxia may lead to endothelial dysfunction, oxidative stress, neuroinflammation, and BBB hyperpermeability, resulting in the development or exacerbation of cognitive deficits [10]. In a breakthrough study, some cortical network events termed paroxysmal slow wave events (PSWEs), which were defined as an EEG median power frequency of < 6 Hz for more than five consecutive seconds, reflected BBB dysfunction, and the occurrence per minute of PSWEs was correlated with levels of cognitive impairment in AD [11]. A broadband reduction in electroencephalography (EEG) spectrum power in a circumscribed regional deficit overlying the parietal cortex is believed to be a useful marker for neural disruption in OSA [12]. However, there have been no studies on the correlation between the PSWEs and cognitive impairment in patients with OSA and the change of PSWEs in patients with OSA after treatment.

In this study, we recruited patients with snoring as the chief complaint, established cross-sectional and longitudinal studies, analyzed PSG recordings from OSA patients, and calculated the occurrence and duration of PSWEs. We put forward a hypothesis that the PSWEs were associated with cognitive impairment in patients with OSA and were related to the levels of cognitive impairment in patients with OSA.

## Materials and methods

### Study design and subjects

This cross-sectional study included participants with subjective snoring complaints recruited from the Sleep Medicine Center of Weihai Municipal Hospital from June 2019 to June 2021. The controls were recruited from community. The inclusion criteria were participants aged 35 to 80 years old without neuropsychological evaluation in the last year and without history of major surgery or trauma in the previous 3 months. No subjects had a previous diagnosis of OSA or dementia, and none had received treatment for snoring.

The exclusion criteria were any previous or current history of neurological or psychiatric disorders that might confuse cognition, including stroke, intracranial infection, traumatic brain injury, hydrocephalus, epilepsy, brain tumors, neurodegenerative diseases (e.g., AD, Parkinson’s disease, multiple system atrophy, and dementia with Lewy bodies), other sleep disorders (e.g., narcolepsy and restless legs syndrome), schizophrenia, major depression, and medication use known to affect cognition or sleep (e.g., benzodiazepines, antihistamines, tricyclic antidepressants, donepezil, and citicoline). Participants were excluded if they were diagnosed with abnormal thyroid function, autoimmune system diseases, pregnancy, severe cardiopulmonary insufficiency, hepatic failure, kidney failure, or cognitive impairment without OSA.

For the longitudinal study, we identified the enrolled OSA patients treated with continuous positive airway pressure (CPAP) and reassessed them after 1 year.

A total of 445 participants conforming to the inclusion criteria were recruited: 15 participants refused to participate in the study, and 72 participants were excluded according to the exclusion criteria. The study and procedures were approved by the same hospital ethics committee. Patients were informed that some of the collected data would be used for research purposes, and informed consent was obtained from all of the subjects before study enrollment.

### Data collection

All of the participants were interviewed to collect information about general health, for instance, age, sex, body mass index (BMI), hypertension, diabetes mellitus, hyperlipidemia, educational attainment, tobacco use (within the previous 3 months), and alcohol consumption (≥ 300 g per week). Serum total cholesterol, low-density lipoprotein cholesterol (LDL-C), triglyceride, and glucose levels were measured.

Hypertension was defined as a blood pressure ≥ 140/90 mmHg in 3 different measurements or current use of antihypertensive drugs. Diabetes mellitus was defined as repeated fasting glucose levels ≥ 126 mg/dl, glucose loads ≥ 200 mg/dl 2 h after oral glucose administration, or current use of antidiabetic medications. Hyperlipidemia was defined as total cholesterol levels ≥ 200 mg/dl, triglyceride levels ≥ 150 mg/dl, LDL-C levels ≥ 130 mg/dl, or the use of lipid-lowering drugs.

### MRI scanning and processing

Brain MRI scans, including head T1-weighted, T2-weighted, T2-weighted fluid-attenuated inversion recovery (FLAIR), and susceptibility-weighted imaging (SWI) sequences, were performed using a 3.0-T Skyra magnetic resonance scanner (Siemens, Erlangen, Germany). FLAIR images were acquired with the following parameters: repetition time (TR)/echo time (TE) = 9000/96 ms; field of view (FOV) = 240 × 240 mm; matrix size = 512 × 512; section thickness = 5 mm. SWI images were acquired with TR = 29 ms, TE = 20 ms, flip angle = 15°, matrix = 448 × 168, FOV = 230 × 173 mm, and slice thickness = 2.0 mm.

The total burden of cerebral small vessel disease (CSVD) was interpreted to assess the severity of CSVD [13]. White matter hyperintensity (WMH) was defined as the presence of hyperintensity in the white matter area. WMH volume was outlined and calculated using the semiautomated freeware 3D-slicer (http://www.slicer.org) [14]. The WMH regions were manually parcellated on template images layer by layer, which were used in automated WMH extraction analysis. All of the scans were reviewed by an experienced neuroradiologist who was blinded to the clinical details.

### Sleep data acquisition and analysis

All of the study subjects underwent overnight in-laboratory PSG (Somté or Grael, Compumedics, 30-40 Flockhart St Abbotsford VIC 3067, Australia) between 22:00 P.M. (lights out) and 6:00 A.M. (lights on) and were asked to abstain from drinking tea and caffeinated beverages during the study.

PSG was conducted using the standard montage recommended by the American Academy of Sleep Medicine (AASM) [15], including EEG channels (F3-M2, F4-M1, C3-M2, C4-M1, T3-M2, T4-M1, O1-M2, and O2-M1), electrooculogram channels, chin electromyogram channels, thermistor and nasal cannula airflow channels, a microphone channel, anterior tibial electromyography, electrocardiography, body position, thoracic and abdominal respiratory movement registration, and a pulse oximetry sensor. EEG data were sampled at 256 Hz. Frequencies less than 0.3 Hz and greater than 70 Hz were filtered, and a notch filter of 50 Hz was applied. All sleep staging and scoring were analyzed strictly by a trained technologist blinded to the clinical information [15, 16].

The PSG parameters evaluated included sleep latency, sleep efficiency, total sleep time (TST), duration of each sleep stage, apnea-hypopnea index (AHI), oxygen desaturation index (ODI) (defined as number of oxygen desaturations ≥3 or 4% per hour), arousal index, mean oxygen saturation during sleep (mean SaO_2_), minimum oxygen saturation during sleep (min SaO_2_), and percentage of sleep time spent with oxygen saturation < 90% (T90). OSA was defined as AHI ≥ 5 events/h, whereas participants with AHI < 5 events/h were regarded as primary snoring subjects. The AHI was used to assess the severity of OSA and was rated as follows: mild ≥ 5 to < 15; moderate ≥ 15 to < 30; and severe ≥ 30 [16].

**Fig. 1:**
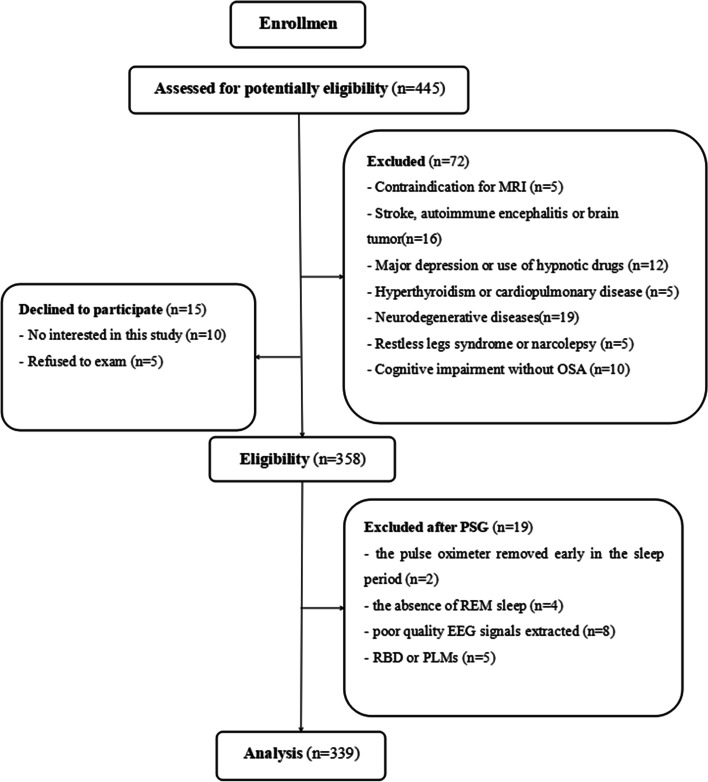
Description of the study population

The subjects were also excluded if the following situations occurred: (1) total sleep time of less than 4 h; (2) rapid eye movement (REM) sleep behavior disorder (RBD) or REM sleep without atonia (RSWA); (3) a large number of periodic limb movements (PLMs) unrelated to respiratory events (total PLM index > 10/h); and (4) REM sleep stage less than 30 min. Nineteen subjects were excluded. Finally, 339 patients were assessed (Fig. 1).

### EEG spectral analyses

**Fig. 2:**
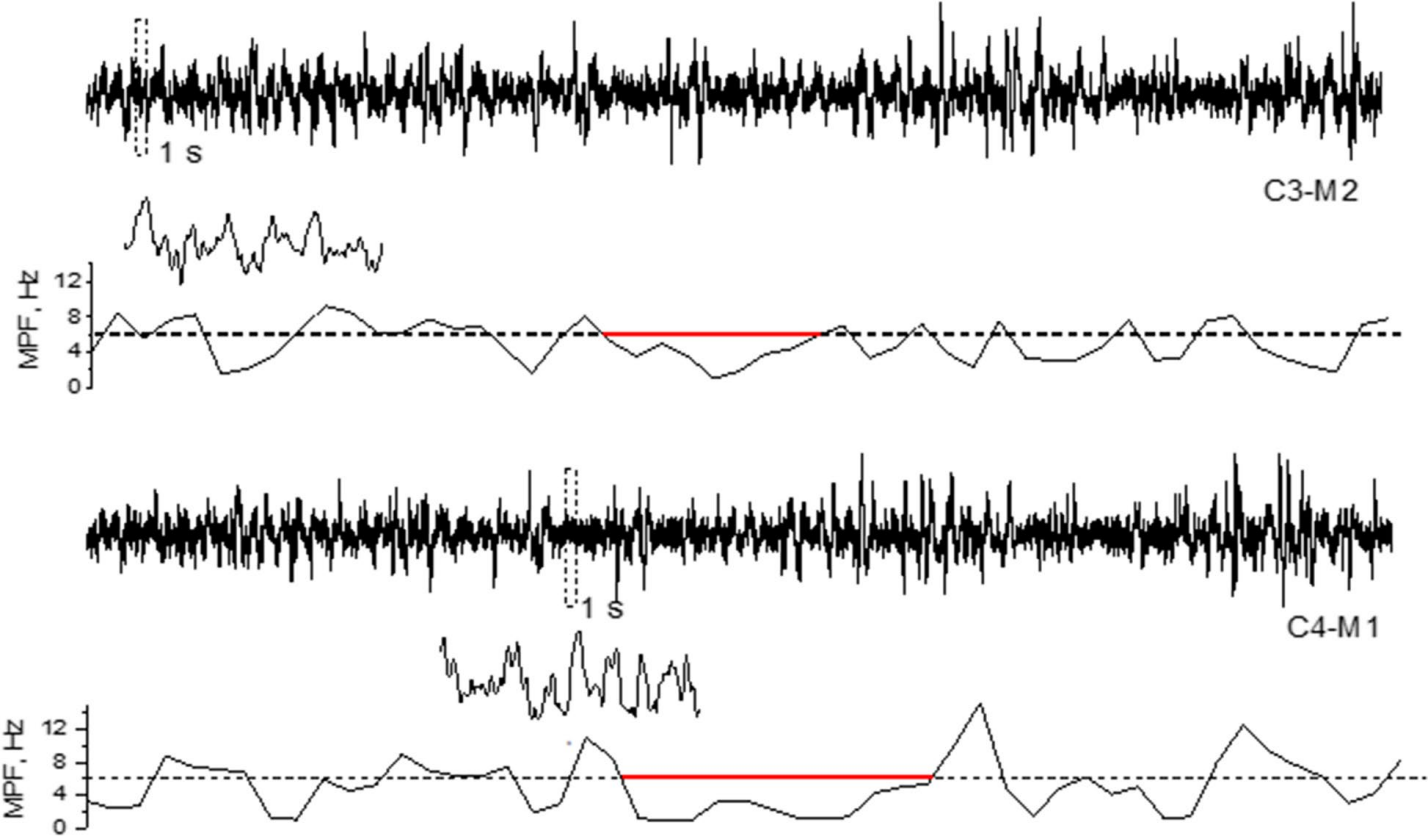
A PSWE detected in a OSA+MCI patient. EEG tracks of C3-M2 and C4-M1 electrodes are shown in the figure. The segment within the dotted rectangle was shown magnified, and those with median power frequency below 6 Hz were plotted on the graph in red

EEG data derived from the wake state (stage W) in the pre-PSG phase and from NREM sleep stage 1 (stage N1), NREM sleep stage 2 (stage N2), NREM sleep stage 3 (stage N3), and REM sleep stage (stage R) were recorded for the spectral analysis. EEG signal processing was performed using self-developed Python scripts based on the research of Milikovsky et al. [11] to detect the occurrence per minute of PSWEs (Fig. 2). PSWE was defined for EEG recordings as a median power frequency of < 6 Hz for more than five consecutive seconds. For our statistical analysis, we focused on the EEG recorded at each channel, and the occurrence per minute of PSWE was calculated separately. Then, we calculated the total PSWEs, which was the sum of the average per-minute multichannel EEG power spectrum for all sleep stages. We also calculated the duration ratios and voltage of PSWEs to fully analyze the characteristics of the PSWEs. The analyzers were blinded to the subjects’ clinical details.

### Neuropsychological and psychopathological evaluation

All of the study subjects completed the Mini-Mental State Examination (MMSE), Montreal Cognitive Assessment (MoCA) Beijing version, Epworth Sleepiness Scale (ESS), Hamilton Anxiety Scale (HAMA), and Hamilton Depression Scale (HAMD)-17 on the subsequent morning following PSG. The neuropsychological tests were administered by the same trained examiner in a quiet room.

We used this MoCA cutoff value (≤ 25 indicating MCI) in agreement with Nasreddine’s study [17] and referred to the revised Mayo Clinic criteria [18] to diagnose MCI, that is (1) subjective cognitive impairment; (2) objective impairment in one or more cognitive domains; (3) slightly impaired complex instrumental daily abilities, but independent daily living abilities; and (4) no dementia. All subjects were divided into three groups based on AHI and MoCA scores: the OSA+MCI group (AHI ≥ 5 and MoCA score ≤ 25); the OSA-MCI group (AHI ≥ 5 and MoCA > 25), and the control group (AHI < 5 and MoCA > 25).

### Exosome extraction and quantification of Aβ42

The fasting blood of all participants was drawn at baseline between 6:00 and 7:00 A.M. and stored in polypropylene tubes containing EDTA. After drawing, the blood samples were centrifuged at 4000×g for 10 min to obtain the plasma. Specific neuron-derived exosomes (NDEs) were isolated according to our published protocol [19]. In brief, using ExoQuick exosome precipitation solution (EXOQ, EXOQ20A-1, System Biosciences, USA), total exosomes were collected from plasma. NDEs were then isolated by coimmunoprecipitation using a rabbit anti-L1 cell adhesion molecule (L1CAM) antibody (eBiosciences, 13-1719-82, San Diego, USA) and labeled with biotin by the EZ-Link sulfo-NHS-biotin system (Thermo Fisher Scientific, 53117, Waltham, MA, USA). Western blotting (WB) and transmission electron microscopy (TEM) were performed to confirm the success of exosome collection. The neural-derived exosome levels of Aβ42 were measured using ELISA kits (Invitrogen, KHB3544, Waltham, MA, USA).

### Enzyme-linked immunosorbent assays of serum CyPA and MMP-9 levels

The serum cyclophilin A (CyPA) and matrix metallopeptidase-9 (MMP-9) levels were detected with ELISA kit (RayBio, 1113202129, Norcross, GA, USA) (RayBio, 0710200173, Norcross, GA, USA) according to our published protocol [20]. All ELISA microplates were read using a Varioskan LUX 3020 instrument (Thermo Fisher Scientific Oy, Ratastie 2, Vantaa, Finland). Measurements were completed by the same technician, who was blinded to the clinical details.

### Intervention

Thirty-eight patients with moderate to severe OSA (AHI≥15 events/h) agreed to be treated by CPAP set at 4–20 cmH_2_O. The inclusion criteria for the longitudinal study were as follows: (1) good compliance with CPAP therapy, using the device for more than 4 h per night and more than 5 nights per week [21]; (2) the effectiveness of CPAP treatment, documented by the ventilator software report (AHI < 5 events/h); and (3) at least 1 year of CPAP therapy. During the CPAP therapy period, 11 patients who discontinued CPAP treatment due to intolerance were excluded. Eventually, 27 patients were identified for analysis.

### Statistical analysis

Continuous variables that conformed to a normal distribution are presented as the means ± standard deviations (SDs), and data not conforming to a normal distribution are reported in the form of medians (interquartile ranges, IQRs). Categorical variables are presented as frequencies (percentages). Statistical significance of differences between means for three groups conforming to a normal distribution was determined with Student’s unpaired *t* test or one-way analyses of variance (ANOVA) with Bonferroni’s post hoc test. The variables with a nonnormal distribution were compared using the nonparametric Mann-Whitney *U* test or Kruskal-Wallis *H* test. Categorical variables were compared using the *χ*^2^ test.

Binary logistic regression analysis was used to investigate the factors influencing MCI in patients with OSA. The linear relationship between the PSWEs in patients with OSA and other factors was analyzed by multiple linear regression analysis. We performed a mediation analysis to test whether the PSWEs mediated the relationship between OSA and cognition. The sleep parameters were considered the independent variable, and MoCA scores were the outcome, while the PSWEs were the mediator. Wilcoxon’s test was used to compare changes in markers of BBB dysfunction before and after treatment. All of the analyses were performed using SPSS 22.0 software (Chicago, IL, USA), R 4.1.1, and GraphPad Prism 5. Statistical significance was defined as *P <* 0.05, and all reported *P* values were two tailed.

## Results

### Demographic and clinical characteristics of the study cohorts

The participants in the study included 229 men and 110 women. A total of 275 patients were diagnosed with OSA, comprising 157 subjects (57.1%) classified as the OSA+MCI group and 118 subjects (42.9%) classified as the OSA-MCI group. The remaining 64 participants were placed in the control group.

The characteristics of all subjects are displayed in Table 1. The differences between the three groups were statistically significant in age, hypertension, hyperlipidemia, glucose levels, serum CyPA levels, serum MMP-9 levels, NDE Aβ_42_ levels, WMH volume, total CSVD burden scores, AHI, arousal index, mean SaO_2_, T90, time ratio of N1, and time ratio of N3 (*P =* 0.027, *P =* 0.004, *P =* 0.028, *P =* 0.001, *P =* 0.006, *P =* 0.003, *P <* 0.001, *P <* 0.001, *P <* 0.001, *P <* 0.001, *P <* 0.001, *P <* 0.001, *P <* 0.001, *P <* 0.001, *P <* 0.001, respectively) by using one-way ANOVA tests, Kruskal-Wallis *H* test, or *χ*^2^ test. Furtherly, the multiple comparisons were carried out separately. All of the above indicators were significantly higher in the OSA+MCI group than in both the OSA-MCI (*P =* 0.029, *P =* 0.026, *P =* 0.002, *P =* 0.031, *P =* 0.011, *P =* 0.016, *P <* 0.001, *P <* 0.001, *P =* 0.011, *P =* 0.018, *P =* 0.007, *P =* 0.025, *P =* 0.002, *P =* 0.020, respectively) and control (*P =* 0.023, *P =* 0.002, *P =* 0.003, *P =* 0.004, *P =* 0.003, *P <* 0.001, *P <* 0.001, *P <* 0.001, *P <* 0.001, *P <* 0.001, *P <* 0.001, *P <* 0.001, *P <* 0.001, *P <* 0.001, respectively) groups except for the proportion of hyperlipidemia. The Aβ42 levels in plasma NDEs and the WMH volume in individuals in the control, OSA-MCI, and OSA+MCI groups gradually increased (OSA+MCI > OSA-MCI > control). The BMI, systolic blood pressure, diastolic blood pressure, ESS scores, ODI, min SaO_2_, and time ratio of REM were statistically different between the three groups (*P <* 0.001, *P =* 0.013, *P =* 0.022, *P <* 0.001, *P <* 0.001, *P <* 0.001, *P <* 0.001, respectively), and were significantly higher in the OSA+MCI (*P <* 0.001, *P =* 0.001, *P =* 0.001, *P <* 0.001, *P <* 0.001, *P <* 0.001, *P <* 0.001, respectively) and OSA-MCI groups (*P <* 0.001, *P =* 0.001, *P <* 0.001, *P <* 0.001, *P <* 0.001, *P <* 0.001, *P =* 0.002, respectively) than those of the control group, but we found no significant differences between the OSA+MCI and OSA-MCI groups. No differences in other clinical features, such as sex, education, diabetes mellitus, HAMA scores, HAMD scores, and TST, were observed among the three groups.

**Table 1:** Univariate analysis of demographic and clinical parameters in three groups (*n*=339)

	OSA+MCI (***n*** = 157)	OSA-MCI (***n*** = 118)	Control (***n*** = 64)	***P*** value
**Demographics**				
Age, years (mean ± SD)	60.32 ± 9.87^a b^	57.36 ± 11.10	57.28 ± 9.18	0.027
Male, *n* (%)	106 (67.52)	85 (72.03)	38 (59.38)	0.219
BMI, kg/m^2^ (mean ± SD)	27.75 ± 3.83^b^	28.04 ± 4.19^b^	25.00 ± 3.71	<0.001
Education, *y*ears, median (IQR)	11 (3)	12 (7)	12 (4)	0.105
**Risk factors**				
Hypertension, *n* (%)	97 (61.78)^a b^	57 (48.31)	25 (39.06)	0.004
Diabetes mellitus, *n* (%)	41 (26.11)	18 (15.25)	11 (18.75)	0.081
Hyperlipidemia, *n* (%)	61 (38.85)^b^	38 (32.20)	13 (20.31)	0.028
Drinking, *n* (%)	44 (28.03)	36 (30.51)	15 (23.44)	0.598
Smoking, *n* (%)	33 (21.02)	31 (26.27)	14 (21.88)	0.575
Systolic blood pressure, mmHg, median (IQR)	140 (27)^b^	137 (23)^b^	132 (25)	0.013
Diastolic blood pressure, mmHg, median (IQR)	85 (15)^b^	85 (13)^b^	80 (13)	0.021
**Laboratory indicators**				
Total cholesterol, mg/dl (mean ± SD)	180.54 ± 45.62	184.41 ± 43.69	182.86 ± 33.25	0.781
Triglycerides, mg/dl, median (IQR)	136.44 (101.00)	136.00 (69.11)	120.50 (73.54)	0.318
LDL-C, mg/dl (mean ± SD)	109.52 ± 33.63	110.57 ± 29.77	109.29 ± 30.16	0.966
Glucose, mg/dl, median (IQR)	99.36 (30.78)^a b^	94.68 (16.20)	93.78 (15.48)	0.001
Serum CyPA, ng/ml (mean ± SD)	8.95 ± 4.49^a b^	7.82 ± 4.00	7.09 ± 3.89	0.006
Serum MMP-9, ng/ml (mean ± SD)	584.63 ± 208.08^a b^	522.22 ± 188.15	494.92 ± 193.04	0.003
NDE Aβ_42_, pg/ml (mean ± SD)	3.18 ± 0.65^a b^	2.98 ± 0.71^b^	2.76 ± 0.65	<0.001
**Neuropsychological test**				
MMSE scores, median (IQR)	27 (4)^a b^	29 (1)	30 (1)	<0.001
MoCA scores, median (IQR)	23 (4)^a b^	27 (2)	27 (3)	<0.001
HAMA scores, median (IQR)	7 (8)	6 (6)	7 (5)	0.297
HAMD scores, median (IQR)	7 (5)	5 (6)	6 (5)	0.077
ESS scores, median (IQR)	9 (6)^b^	8 (6)^b^	5 (6)	<0.001
**Image quantitative measurement**				
WMH volume, mm^3^, median (IQR)	5300.71 (5342.47)^a b^	4222.46 (3029.90)^b^	3275.27 (1787.85)	<0.001
Total CSVD burden scores, median (IQR)	1 (2)^a b^	0.5 (1)	0 (1)	<0.001
**PSG data**				
AHI, events/h, median (IQR)	33.30 (39.30)^a b^	22.60 (37.58)^b^	2.20 (2.48)	<0.001
5≤AHI<15, *n* (%)	32 (20.38)	42 (35.59)	0	
15≤AHI<30, *n* (%)	39 (24.84)	28 (23.73)	0	
30≤AHI, *n* (%)	86 (54.78)	48 (40.68)	0	0.014
ODI, events/h, median (IQR)	26.20 (34.75)^b^	17.90 (27.85)^b^	1.95 (2.18)	<0.001
TST, min (mean ± SD)	354.41 ± 62.52	367.72 ± 58.65	364.67 ± 60.06	0.173
Sleep latency, min, median (IQR)	20.50 (24.00)	19.75 (29.13)	29.50 (34.63)	0.210
Sleep efficiency, %, median (IQR)	74.90 (21.53)	76.90 (18.55)	78.49 (20.72)	0.234
Arousal index, events/h, median (IQR)	20.20 (22.55)^a b^	16.05 (18.70)^b^	6.10 (9.20)	<0.001
Mean SaO_2_, %, median (IQR)	94.00 (3.00)^a b^	95.00 (2.25)^b^	96.00 (2.00)	<0.001
Min SaO_2_, %, median (IQR)	82.00 (12.50)^b^	82.50 (13.00)^b^	90.00 (4.00)	<0.001
T90, % , median (IQR)	4.60 (13.65)^a b^	2.45 (9.65)^b^	0.00 (0.20)	<0.001
NREM stage 1 of TST, %, median (IQR)	15.80 (8.65)^a b^	13.00 (9.03)^b^	9.35 (4.98)	<0.001
NREM stage 2 of TST, % (mean ± SD)	52.09 ± 8.89	52.58 ± 8.20	51.84 ± 7.05	0.821
NREM stage 3 of TST, %, median (IQR)	15.60 (8.40)^a b^	17.75 (9.42)^b^	20.15 (8.20)	<0.001
REM stage of TST, % (mean ± SD)	14.21 ± 4.62^b^	15.07 ± 4.77^b^	17.50 ± 5.20	<0.001

1mmHg = 0.133kPa

*Abbreviations*: *OSA* obstructive sleep apnea, *MCI* mild cognitive impairment, *SD* standard deviation, *IQR* interquartile range, *BMI* body mass index, *LDL-C* low-density lipoprotein cholesterol, *MMSE* Mini-Mental State Examination, *MoCA* Montreal Cognitive Assessment, *ESS* Epworth Sleepiness Scale, *HAMA* Hamilton Anxiety Scale, *HAMD* Hamilton Depression Scale, *WMH* white matter hyperintensity, *CSVD* cerebral small vessel disease, *CyPA* cyclophilin A, *MMP-9* matrix metallopeptidase-9, *Aβ* amyloid-β, *AHI* apnea-hypopnea index, *ODI* oxygen desaturation index, *TST* total sleep time, *NREM* nonrapid eye movement, *REM* rapid eye movement, *mean SaO*_*2*_ mean oxygen saturation during sleep, *min SaO*_*2*_ minimum oxygen saturation during sleep, *T90* percentage of time oxygen saturation <90%, *PSWE* paroxysmal slow wave event

^a^*P*<0.05 compared with OSA-MCI

^b^*P*<0.05 compared with control

### PSWEs in OSA+MCI patients, OSA-MCI patients, and control subjects

**Fig. 3:**
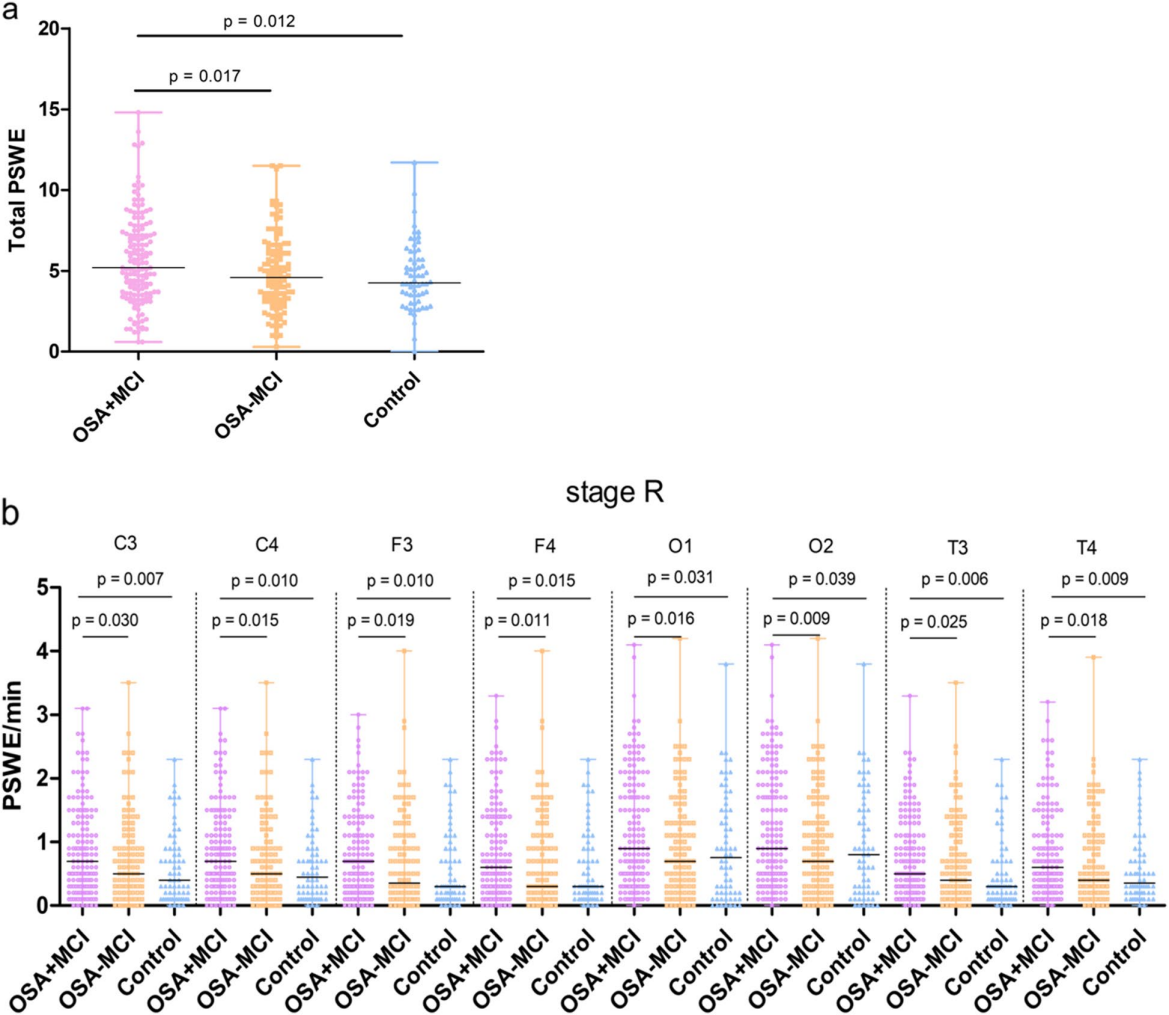
The occurrence of PSWEs in the cross-sectional OSA+MCI, OSA-MCI, and control groups. **a** The occurrence of the total PSWEs in OSA+MCI group was higher than that in OSA-MCI and control groups. **b** The occurrence per minute of the PSWEs in stage R in OSA+MCI group were higher than those in the OSA-MCI and control groups, regardless of the EEG electrode

As shown in Fig. 3, when the individual groups were analyzed by the nonparametric Mann-Whitney *U* test, the occurrence of the total PSWEs in the OSA+MCI group was higher than that in the OSA-MCI [5.20 (3.70) vs 4.60 (3.05), *P =* 0.017] and control groups [5.20 (3.70) vs 4.25 (2.50), *P =* 0.012) (Fig. 3a). Furthermore, the occurrence per minute of PSWEs for stage R in the OSA+MCI group was higher than that in the OSA-MCI [C3, 0.70 (1.05) vs 0.50 (0.83), *P =* 0.030; C4, 0.70 (1.00) vs 0.50 (0.70), *P =* 0.015; F3, 0.70 (0.95) vs 0.35 (0.83), *P =* 0.019; F4, 0.60 (1.10) vs 0.30 (0.83), *P =* 0.011; T3, 0.50 (0.80) vs 0.40 (0.83), *P =* 0.025; T4, 0.60 (0.80) vs 0.40 (0.80), *P =* 0.018; O1, 0.90 (1.45) vs 0.70 (1.03), *P =* 0.016; O2, 0.90 (1.45) vs 0.70 (1.10), *P =* 0.009] and control [C3, 0.70 (1.05) vs 0.40 (0.80), *P =* 0.007; C4, 0.70 (1.00) vs 0.45 (0.68), *P =* 0.010; F3, 0.70 (0.95) vs 0.30 (0.80), *P =* 0.010; F4, 0.60 (1.10) vs 0.30 (0.70), *P =* 0.015; T3, 0.50 (0.80) vs 0.30 (0.60), *P =* 0.006; T4, 0.60 (0.80) vs 0.35 (0.60), *P =* 0.009; O1, 0.90 (1.45) vs 0.75 (1.30), *P =* 0.031; O2, 0.90 (1.45) vs 0.80 (1.38), *P =* 0.039] groups, regardless of the location of the scalp electrode (Fig. 3b). Nevertheless, there were no significant differences in the PSWEs for stages N1, N2, N3, and W among the OSA+MCI, OSA-MCI, and control groups (all *P >* 0.05).

**Fig. 4:**
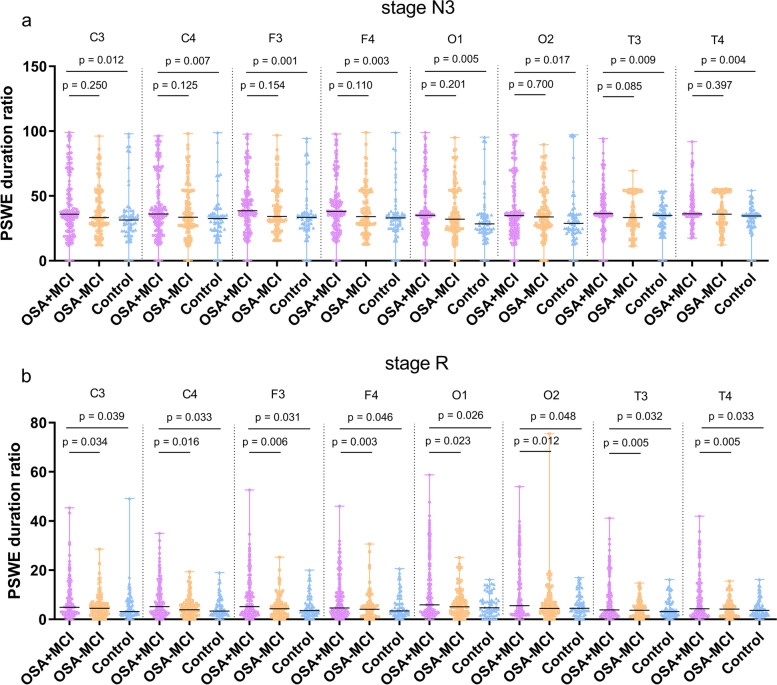
The duration ratios of PSWEs in the cross-sectional OSA+MCI, OSA-MCI, and control groups. **a** The duration ratios of the PSWEs in stage N3 in OSA+MCI group were higher than those in the control groups, regardless of the EEG electrode. **b** The duration ratios of the PSWEs in stage R in OSA+MCI group were higher than those in the OSA-MCI and control groups, regardless of the EEG electrode

As shown in Fig. 4, the duration ratios of the PSWEs for stage N3 in the OSA+MCI group were higher than those in the control [C3, 35.98 (27.40)% vs 31.47 (14.51)%, *P =* 0.012; C4, 36.10 (28.54)% vs 32.59 (13.42)%, *P =* 0.007; F3, 38.53 (20.39)% vs 33.45 (12.12)%, *P =* 0.001; F4, 38.02 (22.16)% vs 33.05 (12.12)%, *P =* 0.003; T3, 36.31 (12.31)% vs 34.91 (13.12)%, *P =* 0.009; T4, 36.18 (13.47)% vs 34.60 (8.6)%, *P =* 0.004; O1, 35.05 (25.71)% vs 28.44 (12.88)%, *P =* 0.005; O2, 34.87 (30.93)% vs 28.85 (13.31)%, *P =* 0.017], but we found no significant differences between the OSA+MCI and OSA-MCI groups or between the OSA-MCI and control groups (Fig. 4a). The duration ratios of the PSWEs for stage R in the OSA+MCI group were higher than those in the OSA-MCI [C3, 4.87 (8.02)% vs 4.49 (4.42)%, *P =* 0.034; C4, 5.15 (8.45)% vs 3.88 (4.38)%, *P =* 0.016; F3, 5.16 (8.53)% vs 4.33 (4.88)%, *P =* 0.006; F4, 4.60 (8.48)% vs 4.11 (4.49)%, *P =* 0.003; T3, 3.86 (8.17)% vs 3.73 (4.30)%, *P =* 0.005; T4, 4.27 (9.54)% vs 4.17 (4.64)%, *P =* 0.005; O1, 5.91 (13.40)% vs 5.12 (5.88)%, *P =* 0.023; O2, 5.58 (13.73)% vs 4.43 (4.96)%, *P =* 0.12] and control [C3, 4.87 (8.02)% vs 3.17 (5.54)%, *P =* 0.039; C4, 5.15 (8.45)% vs 3.34 (5.65)%, *P =* 0.033; F3, 5.16 (8.53)% vs 3.57 (6.03)%, *P =* 0.031; F4, 4.60 (8.48)% vs 3.42 (6.07)%, *P =* 0.046; T3, 3.86 (8.17)% vs 3.17 (5.04)%, *P =* 0.032; T4, 4.27 (9.54)% vs 3.60 (4.29)%, *P =* 0.033; O1, 5.91 (13.40)% vs 4.69 (5.89)%, *P =* 0.026; O2, 5.58 (13.73)% vs 4.50 (6.57)%, *P =* 0.048] groups, regardless of the location of the scalp electrode (Fig. 4b). However, there were no significant differences in the PSWE duration ratios for stages N1, N2, and W among the OSA+MCI, OSA-MCI, and control groups (all *P >* 0.05), and we did not find statistically significant differences in the voltage of PSWEs between the three groups (all *P >* 0.05).

### Adjusted relationship between PSWEs and MCI in patients with OSA

In addition, binary logistic regression analysis revealed that the total PSWEs (odds ratio (OR) 1.113, 95% confidence intervals (CIs) 1.007–1.229, *P =* 0.035) and the occurrence per minute of the PSWEs at the C4-M1 (OR 1.446, 95%CIs 1.004–2.081, *P* = 0.048), O1-M2 (OR 1.409, 95% CIs 1.043–1.904, *P =* 0.025), and O2-M1 (OR 1.461, 95% CIs 1.084–1.968, *P =* 0.013) channels for stage R were independent influence factors of cognitive impairment in patients with OSA when adjusting for age, sex, BMI, and education. The total PSWEs (OR 1.117, 95% CIs 1.005-1.242, *P =* 0.040) and the occurrence per minute of the PSWEs at the F4-M1 (OR 1.451, 95% CIs 1.004–2.096, *P =* 0.048), O1-M2 (OR 1.426, 95% CIs 1.039–1.957, *P =* 0.028), and O2-M1 (OR 1.470, 95% CIs 1.075–2.010, *P =* 0.016) channels for stage R were independent influence factors of cognitive impairment in patients with OSA when adjusting for age, sex, education, vascular risk factors (e.g., hypertension, diabetes, hyperlipidemia), HAMD scores, Aβ42, and AHI, indicating increased odds of being cognitively impaired with higher PSWEs in patients with OSA (Tables 2 and 3).

**Table 2:** Logistic regression analysis between the PSWEs and MCI in patients with OSA (*n*=275)

	Model 0		Model 1		Model 2	
	OR (95%CIs)	***P*** value	OR (95%CIs)	***P*** value	OR (95%CIs)	***P*** value
Total PSWEs	1.130 (1.026–1.244)	0.013	1.115 (1.009–1.231)	0.032	1.119 (1.007–1.245)	0.037
The PSWEs at C3/M2 in stage R	1.385 (0.973–1.973)	0.071	1.394 (0.975–1.993)	0.069	1.363 (0.944–1.969)	0.099
The PSWEs at C4/M1 in stage R	1.445 (1.008–2.072)	0.045	1.452 (1.009–2.090)	0.045	1.436 (0.985–2.095)	0.060
The PSWEs at F3/M2 in stage R	1.360 (0.950–1.948)	0.093	1.370 (0.952–1.973)	0.090	1.389 (0.951–2.027)	0.089
The PSWEs at F4/M1 in stage R	1.424 (1.006–2.016)	0.046	1.432 (1.005–2.039)	0.047	1.454 (1.006–2.098)	0.046
The PSWEs at O1/M2 in stage R	1.415 (1.055–1.900)	0.021	1.409 (1.043–1.902)	0.025	1.428 (1.041–1.959)	0.027
The PSWEs at O2/M1 in stage R	1.460 (1.091–1.954)	0.011	1.457 (1.082–1.962)	0.013	1.470 (1.076–2.010)	0.016
The PSWEs at T3/M2 in stage R	1.382 (0.942–2.027)	0.098	1.377 (0.933–2.032)	0.107	1.373 (0.918–2.053)	0.123
The PSWEs at T4/M1 in stage R	1.422 (0.981–2.062)	0.063	1.407 (0.964–2.053)	0.077	1.397 (0.944–2.069)	0.095

Model 0: unadjusted

Model 1: adjusted for age, sex, BMI, and education

Model 2: adjusted for age, sex, education, vascular risk factors (e.g., hypertension, diabetes mellitus, hyperlipidemia), HAMD scores, Aβ42, and AHI

*Abbreviations*: *PSWE* paroxysmal slow wave event, *MCI* mild cognitive impairment, *OSA* obstructive sleep apnea, *OR* odds ratio, *CIs* confidence interval, *HAMD* Hamilton Depression Scale, *Aβ* amyloid-β, *AHI* apnea-hypopnea index, *R* rapid eye movement

**Table 3:** Logistic regression analysis between the PSWEs and MCI in patients with OSA (*n*=275)

	Model 0		Model 1	
	OR (95%CIs)	***P*** value	OR (95%CIs)	***P*** value
Duration ratios of PSWEs at C3/M2 in stage R	1.067 (1.020–1.117)	0.005	1.058 (1.009–1.109)	0.019
Duration ratios of PSWEs at C4/M1 in stage R	1.090 (1.038–1.145)	0.001	1.091 (1.034–1.151)	0.001
Duration ratios of PSWEs at F3/M2 in stage R	1.084 (1.038–1.132)	<0.001	1.084 (1.033–1.137)	0.001
Duration ratios of PSWEs at F4/M1 in stage R	1.078 (1.033–1.124)	<0.001	1.074 (1.026–1.124)	0.002
Duration ratios of PSWEs at O1/M2 in stage R	1.062 (1.030–1.096)	<0.001	1.063 (1.027–1.099)	<0.001
Duration ratios of PSWEs at O2/M1 in stage R	1.060 (1.027–1.094)	<0.001	1.058 (1.022–1.095)	0.001
Duration ratios of PSWEs at T3/M2 in stage R	1.108 (1.052–1.166)	<0.001	1.109 (1.048–1.174)	<0.001
Duration ratios of PSWEs at T4/M1 in stage R	1.102 (1.049–1.158)	<0.001	1.105 (1.046–1.166)	<0.001

Model 0: unadjusted

Model 1: adjusted for age, sex, education, vascular risk factors (e.g., hypertension, diabetes mellitus, hyperlipidemia), HAMD scores, Aβ42, and AHI

*Abbreviations*: *PSWE* paroxysmal slow wave event, *MCI* mild cognitive impairment, *OSA* obstructive sleep apnea, *OR* odds ratio, *CIs* confidence interval, *HAMD* Hamilton Depression Scale, *Aβ* amyloid-β, *AHI* apnea-hypopnea index, *R* rapid eye movement

The duration ratios of the PSWEs at the C3-M2 (OR 1.057, 95% CIs 1.009–1.108, *P =* 0.020), C4-M1 (OR 1.090, 95% CIs 1.033–1.150, *P =* 0.002), F3-M2 (OR 1.083, 95% CIs 1.033–1.136, *P =* 0.001), F4-M1 (OR 1.073, 95% CIs 1.025–1.123, *P =* 0.002), O1-M2 (OR 1.062, 95% CIs 1.027–1.099, *P* < 0.001), O2-M1 (OR 1.058, 95% CIs 1.022–1.095, *P =* 0.001), T3-M2 (OR 1.108, 95% CIs 1.047–1.173, *P* < 0.001), and T4-M1 (OR 1.104, 95% CIs 1.045–1.166, *P* < 0.001) channels for stage R were independent influence factors of cognitive impairment in patients with OSA when adjusting for age, sex, education, vascular risk factors (e.g., hypertension, diabetes, hyperlipidemia), HAMD scores, Aβ42, and AHI, indicating that a higher duration of PSWEs for stage R was independently associated with the higher risk of cognitive impairment in patients with OSA.

### Relative independent factors affecting PSWEs in patients with OSA

Multivariable linear regression analysis was performed (Table 4). We found that WMH volume, ESS scores, mean SaO_2_, and T90 had a linear effect on the total PSWEs (*β* 0.207, standard error (SE) 0.000, *P* = 0.001; *β* 0.138, SE 0.034, *P* = 0.023; *β* −0.355, SE 0.048, *P* < 0.001; *β* 0.130, SE 0.009, *P* = 0.029, respectively) when adjusting for age, sex, and vascular risk factors (e.g., hypertension, diabetes, hyperlipidemia). Similar to the total PSWE findings, the mean SaO_2_ had a significant, negative effect on the occurrence per minute of PSWEs in the F4-M1, O1-M2, and O2-M1 channels in stage R (*β* −0.229, SE 0.014, *P* < 0.001; *β* −0.175, SE 0.017, *P* = 0.004; *β* −0.175, SE 0.017, *P* = 0.004, respectively), but no effect of WMH volume, total CSVD burden, or ESS was found.

**Table 4:** Multivariable linear regression analysis between the PSWEs and various indicators in patients with OSA (*n*=275)

	Total PSWEs		PSWEs at F4/M1 in stage R		PSWEs at O1/M2 in stage R		PSWEs at O2/M1 in stage R	
	***β*** (SE)	***P*** value	***β*** (SE)	***P*** value	***β*** (SE)	***P*** value	***β*** (SE)	***P*** value
NDE Aβ_42_	0.106 (0.228)	0.076	0.047 (0.065)	0.440	0.060 (0.075)	0.323	0.063 (0.076)	0.296
WMH volume	0.206 (0.000)	0.001	0.039 (0.000)	0.527	0.089 (0.000)	0.146	0.066 (0.000)	0.281
Total CSVD burden	0.087 (0.144)	0.137	0.031 (0.041)	0.606	0.078 (0.049)	0.189	0.089 (0.050)	0.133
ESS	0.136 (0.034)	0.025	0.076 (0.010)	0.222	0.036 (0.011)	0.558	0.018 (0.011)	0.763
AHI	0.037 (0.007)	0.546	0.076 (0.002)	0.221	0.035 (0.002)	0.564	0.031 (0.002)	0.618
ODI	−0.015 (0.007)	0.809	0.045 (0.002)	0.464	−0.001 (0.002)	0.989	−0.005 (0.002)	0.941
Mean SaO_2_	−0.351 (0.048)	<0.001	−0.229 (0.014)	<0.001	−0.174 (0.017)	0.004	−0.174 (0.017)	0.004
Min SaO_2_	−0.069 (0.014)	0.263	−0.105 (0.004)	0.094	−0.008 (0.005)	0.897	−0.011 (0.005)	0.855
T90	0.129 (0.009)	0.031	0.122 (0.003)	0.046	0.089 (0.003)	0.141	0.087 (0.003)	0.151

*Abbreviations*: *PSWE* paroxysmal slow wave event, *OSA* obstructive sleep apnea, *SE* standard error, *Aβ* amyloid-β, *WMH* white matter hyperintensity, *CSVD* cerebral small vessel disease, *ESS* Epworth Sleepiness Scale, *AHI* apnea-hypopnea index, *ODI* oxygen desaturation index, *mean SaO*_*2*_ mean oxygen saturation during sleep, *min SaO*_*2*_ minimum oxygen saturation during sleep, *T90* percentage of time oxygen saturation <90%

### Association between PSWEs and serum CyPA and MMP-9 levels

Additionally, Spearman’s correlation coefficients were used to verify the correlation between the PSWEs and serological indicators associated with the breakdown of the BBB (serum CyPA and MMP-9 levels) in patients with OSA (*n* = 275). Positive correlations were identified between the total PSWEs and serum CyPA and MMP-9 levels (*r =* 0.405, *P <* 0.001; *r =* 0.321, *P <* 0.001), but weakly. There were positive but weak correlations between the PSWEs at F4-M1, O1-M2, and O2-M1 in stage R and serum CyPA (*r =* 0.214, *P <* 0.001; *r =* 0.267, *P <* 0.001; *r =* 0.290, *P <* 0.001, respectively) and MMP-9 (*r =* 0.159, *P =* 0.008; *r =* 0.125, *P =* 0.038; *r =* 0.153, *P =* 0.011, respectively) levels.

### Correlation between PSWEs and cognitive performance in patients with OSA

Moreover, we observed a weak negative correlation between the total PSWEs and global cognitive function (described using MoCA scores) (*r =* −0.310, *P <* 0.001) using Spearman’s correlation coefficients. Higher scores indicated better cognitive performance and lower scores, in contrast, poorer cognitive function. We also found that the total PSWEs had a weak negative correlation with all of the cognitive subdomains, including visuoexecutive function, naming, attention, language, abstraction, delayed recall and orientation (*r =* −0.212, *P <* 0.001; *r =* −0.291, *P <* 0.001; *r =* −0.168, *P =* 0.005; *r =* −0.205, *P =* 0.001; *r =* −0.153, *P =* 0.011; *r =* −0.223, *P <* 0.001; *r =* −0.259, *P <* 0.001, respectively). There were negative correlations between the PSWEs at F4-M1, O1-M2, and O2-M1 in stage R and cognitive performance, but the correlations were weak. The details are provided in Table 5.

**Table 5:** Correlation analysis between the PSWEs and cognitive performance in patients with OSA (*n*=275)

	Total PSWEs		PSWEs at F4/M1 in stage R		PSWEs at O1/M2 in stage R		PSWEs at O2/M1 in stage R	
	***r***	***P*** value	***r***	***P*** value	***r***	***p*** value	***r***	***P*** value
Total MoCA scores	−0.310	<0.001	−0.201	0.001	−0.203	0.001	−0.208	0.001
Visuoexecutive	−0.212	<0.001	−0.128	0.034	−0.160	0.008	−0.190	0.002
Naming	−0.291	<0.001	−0.200	0.001	−0.196	0.001	−0.184	0.002
Attention	−0.168	0.005	−0.133	0.028	−0.087	0.151	−0.079	0.190
Language	−0.205	0.001	−0.140	0.020	−0.125	0.038	−0.146	0.016
Abstraction	−0.153	0.011	−0.125	0.039	−0.072	0.234	−0.079	0.194
Delayed recall	−0.223	<0.001	−0.086	0.154	−0.125	0.039	−0.119	0.048
Orientation	−0.259	<0.001	−0.171	0.004	−0.184	0.002	−0.179	0.003

*Abbreviations*: *PSWE* paroxysmal slow wave event, *OSA* obstructive sleep apnea, *MoCA* Montreal Cognitive Assessment

**Fig. 5:**
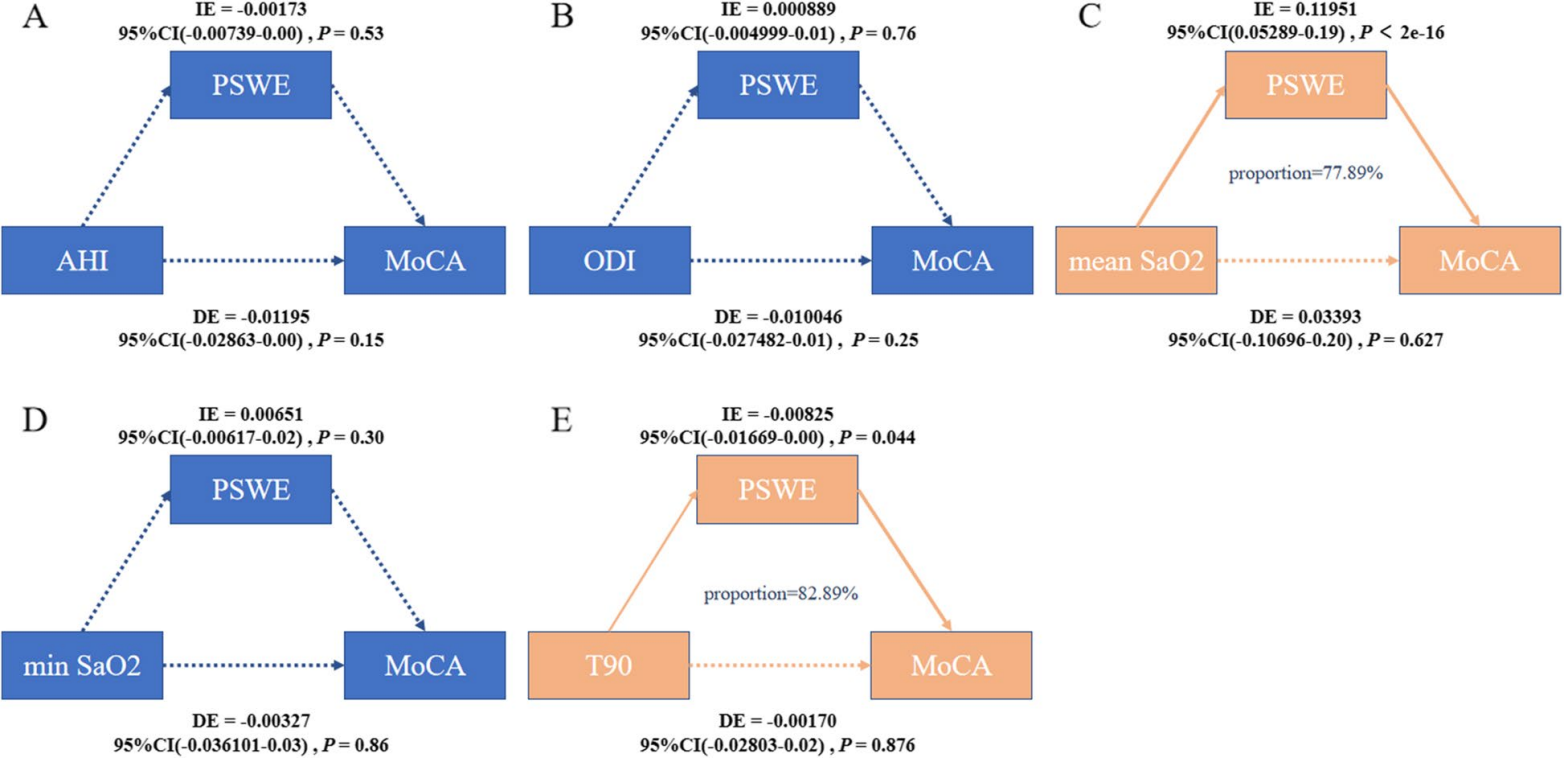
Mediation analyses of the association between sleep characteristics and cognitive impairment in patients with OSA. The relationship between mean SaO_2_ and T90 with MoCA scores was mediated by the total PSWEs

The mediation analysis showed that the relationship between mean SaO_2_ and T90 with MoCA scores was mediated by the total PSWEs after adjusting for age, sex, and education. The effect was considered partial mediation with the proportion of mediation 77.89 and 82.89%. There is no evidence that AHI, ODI, arousal index, min SaO_2_, and ESS contributes to cognitive impairments via modulating PSWEs (Fig. 5).

### Alterations in the occurrence of PSWEs and serum CyPA and MMP-9 levels after CPAP intervention

Among 27 moderate to severe OSA patients who were treated by CPAP and identified for longitudinal analysis, 18 patients had a clinical diagnosis of MCI. After 1 year of CPAP treatment, we observed that ESS scores (5.63 ± 1.74 vs 11.70 ± 2.74, *P <* 0.001), serum CyPA levels (9.30 ± 2.63 ng/ml vs 10.42 ± 3.79 ng/ml, *P =* 0.037), and serum MMP-9 levels (528.94 ± 128.01 ng/ml vs 581.53 ± 201.74 ng/ml, *P =* 0.040) decreased significantly, and MoCA scores (24.44 ± 3.06 vs 24.04 ± 3.36, *P =* 0.039) were improved compared with baseline. Most remarkably, the total PSWEs after CPAP treatment were significantly lower than those at baseline [3.1 (2.5) vs 3.5 (3.2), *P <* 0.001], and the occurrence per minute of PSWEs in stage R was also significantly reduced at either EEG electrode (*P =* 0.033, *P =* 0.043, *P =* 0.026, *P =* 0.017, *P =* 0.040, *P =* 0.044, *P =* 0.022, *P =* 0.028, respectively) (Table 6) but not in stages N1, N2, N3, and W.

**Table 6:** Changes of the PSWEs in OSA patients before and after CPAP treatment (*n*=27)

	Baseline	Follow-up	***P*** value
**Total PSWEs**	3.5 (3.2)	3.1 (2.5)	<0.001
**PSWEs in stage R**			
C3/M2, median (IQR)	0.4 (0.9)	0.4 (0.7)	0.033
C4/M1, median (IQR)	0.3 (0.6)	0.3 (0.6)	0.043
F3/M2, median (IQR)	0.3 (0.9)	0.3 (0.6)	0.026
F4/M1, median (IQR)	0.3 (0.8)	0.3 (0.6)	0.017
O1/M2, median (IQR)	1.0 (1.3)	0.6 (0.7)	0.040
O2/M1, median (IQR)	1.0 (1.2)	0.7 (0.7)	0.044
T3/M2, median (IQR)	0.3 (0.6)	0.3 (0.6)	0.022
T4/M1, median (IQR)	0.3 (0.7)	0.4 (0.5)	0.028

*Abbreviations*: *OSA* obstructive sleep apnea, *CPAP* continuous positive airway pressure, *PSWE* paroxysmal slow wave event, *IQR* interquartile range, *stage R* rapid eye movement sleep stage

## Discussion

In this research, we found some interesting results: (1) the total PSWEs in the OSA+MCI group were higher than those in the OSA-MCI and control groups, and the occurrence per minute and duration ratio of PSWEs in stage REM in the OSA+MCI group significantly increased in frontal, parietal, temporal, and occipital regions; (2) the total PSWEs were independent influencing factors of cognitive impairment in patients with OSA and were positively correlated with serum CyPA and MMP-9 levels, while the total PSWEs had a significantly negative correlation with global cognitive function and all of the cognitive subdomains; (3) the relationship between mean SaO_2_ and T90 with MoCA scores was mediated by the total PSWEs; and (4) the occurrence of total PSWEs and PSWEs in stage REM after 1 year of CPAP intervention was significantly lower than at baseline.

A growing body of evidence has linked OSA to cognitive impairment. Chronic intermittent hypoxia, carbon dioxide retention, and sleep fragmentation in patients with OSA not only increases oxidative stress but also could activate oxygen sensors, perpetuate the state of chronic inflammation, induce neurological imbalance, increased blood pressure, and endocrine disturbance, and affect BBB permeability, thereby altering neuronal morphology and synaptic plasticity and leading to cognitive impairment [2, 22–24], involving a variety of mechanisms, including endothelial dysfunction, oxidative stress, microglia activation and neuroinflammation, impaired sympathetic tone, deregulated glucose homeostasis, and hypertension [23, 25]. BBB dysfunction can be difficult to diagnose because of high cost or invasiveness, so it is urgent to find a simple and easy-to-implement examination method. Milikovsky [11] et al. proposed that PSWEs represent a transient pathological transformation of activity within cortical networks. They identified that the occurrence per minute of PSWEs is correlated with the severity of cognitive impairment in AD, reflecting that destruction of BBB integrity causes neural dysfunction. They also noted a difference in PSWEs; in patients with AD, PSWEs were recorded bilaterally, and in patients with epilepsy, PSWEs were most often focal. A study also showed that the increase in relative theta power might be the first change in patients with dementia due to AD, and the relative theta band was correlated with working memory [26]. Several studies supported that the cyclic alternating pattern (CAP) slow components may play a role in sleep-related cognitive processes [27] and showed an inverse relationship between OSA severity and CAP [28]. It has been emphasized that hypoxia, rather than sleep fragmentation, can cause brain dysfunction with the consequent EEG slowing seen in OSA patients [29]. Some studies indicate that OSA was significantly associated with EEG slowing predominantly in REM sleep rather than NREM sleep, and greater EEG slowing during REM sleep was associated with cognitive decline of OSA patients [30, 31]. Anatomically, certain regions of the brain, such as the frontal lobes, are particularly vulnerable to hypoxic-ischemic injury [32]. More severe nocturnal hypoxia was associated with increased activation during the working memory task in the occipital cortex in participants with OSA [33]. We stated that total PSWEs were significantly increased in OSA patients with MCI compared with OSA patients without cognitive impairment, and the occurrence per minute and duration ratio of PSWEs for stage REM in the OSA+MCI group was also higher. The PSWEs recorded bilaterally in the OSA+MCI group were generally increasing, as we showed, supporting that BBB damage in OSA patients with cognitive impairment is diffuse but not focal. When the PSWE data were analyzed separately for each sleep stage, the results revealed that significant differences only occurred in stage REM, which might have been due to possibly non-persistent EEG changes in the early stages of cognitive impairment, with a relatively prolonged sleep apnea, lower oxygen saturation, and higher heart rate and blood pressure during REM sleep, perhaps leading to a more evident oxidative stress and neuroinflammatory response in this phase. There is an increased vulnerability to the pathophysiological consequences of obstructive events, including hypoxemia, hypercapnia, and oxidative stress, that occur during REM sleep [31]. Unexpectedly, there was no significant difference between the occurrence of PSWEs in the OSA-MCI group and those in the control group, but there was an upward trend. If the sample size of the study were increased, a positive conclusion might be obtained. Our findings indicate that the total PSWEs and PSWEs at frontal and occipital region in stage REM were independently associated with cognitive impairment in OSA patients. The preferential effect on frontal and occipital cortex by PSWE is unexplained, but could be related to the substantial convergence of fiber pathways on the frontal lobe and the role of the occipital lobe as a neurophysiological source of long-range cortical connectivity.

Currently, the CyPA-MMP-9 signaling pathway is presumed to be involved in regulating BBB permeability, because its activation may lead to degradation of tight junctions and basement membrane proteins, resulting in BBB damage [34–36]. In our previous study, we found that increased serum CyPA and MMP-9 levels are associated with MCI in OSA patients and directly related to the severity of WMHs and CSVD [20]. After enlarging the sample size, we still got the same results that the serum CyPA and MMP-9 levels were higher in the OSA+MCI group, implying the same role of the CyPA-MMP-9 pathway in OSA-induced BBB dysfunction. The correlation analysis demonstrated positive correlations between the total PSWEs and serum CyPA and MMP-9 levels in the present cohort, which also supported PSWEs’ ability to reflect BBB dysfunction, indicating that the effect of BBB dysfunction on cognitive impairment in patients with OSA. Additionally, we further observed that mean SaO_2_ had a detrimental effect on PSWEs, suggesting that the occurrence of PSWEs was significantly increased by hypoxia but not by AHI. It is worth noting that the relationship between the mean oxygen saturation and the percentage of time with oxygen saturation less than 90% during sleep with MoCA scores was mediated by the total PSWEs. Collectively, BBB dysfunction in patients with OSA was more aggravated with hypoxia, rather than the severity of OSA, leading to cognitive impairment. Like other studies [37], we also found that cognitive performance reflected by MoCA scores in patients with OSA was improved after CPAP intervention. After CPAP treatment, the total PSWEs, PSWEs in stage REM, and serum CyPA and MMP-9 levels all decreased significantly, confirming that BBB dysfunction in OSA patients may be partially reversible and CPAP therapy has considerable therapeutic value for BBB dysfunction in OSA patients.

Exosomes derived from the central nervous system can pass through the BBB and exist in the peripheral blood [38], and a study verified the agreement between CSF and blood exosomal biomarkers [39]. Aβ protein, one of the classical AD biomarkers, is secreted in exosomes during their formation in the brain [40]. BBB dysfunction triggers neuroinflammation and oxidative stress and then promotes Aβ generation, giving rise to cognitive impairment and the onset of dementia [41]. Previous studies have demonstrated that OSA could impair Aβ clearance and affect the relationship between slow wave activity and Aβ [42]. Hypoxia results in increased production and decreased clearance of Aβ [43, 44]. As OSA severity increases, the burden of Aβ increases [45]. Our results showed that Aβ42 levels in plasma NDEs were gradually elevated in control, OSA-MCI, and OSA+MCI patients, not contradicting the previous research [42, 45, 46]. Our study demonstrated that the higher the occurrence and duration of PSWEs for stage REM in patients with OSA, the higher the risk of cognitive impairment, independent of Aβ, consistent with previous studies suggesting that BBB breakdown is an early pathogenic event contributing to neurodegeneration in AD and MCI independent of classic AD biomarkers [7]. We found that WMH volume not the total burden of CSVD had a positive and linear effect on total PSWEs. Current evidence suggests that moderate to severe sleep apnea is positively related to WMH [47]. BBB damage might cause structural changes in cerebral microvascular disease, so BBB permeability is increased with worsening WMH [48, 49], and higher BBB permeability is associated with greater WMH burden and cognitive decline [50].

## Limitations

Limitations of this study should be addressed. First, because the inclusion criteria allowed for snoring patients, there was selection bias in the study population, resulting in too small sample sizes of the participants without OSA and the possibility of residual confounding. The replication and validation of the results in a larger community sample, including a group with MCI but no OSA, are required. Second, because of the fragmented sleep of OSA patients, we did not choose to analyze the EEG of the entire night but only a fragment of different sleep periods. Finally, due to insufficient patient compliance, few patients received CPAP treatment, affecting the evaluation of outcomes.

## Conclusions

In summary, the occurrence and duration of PSWEs were increased in OSA patients with cognitive impairment. The PSWEs were implicated in cognitive impairment and its levels in patients with OSA, and odds of being cognitively impaired were increased with the increased occurrence of PSWEs. Our study revealed that hypoxia in OSA patients can lead to cognitive impairment through BBB dysfunction. After 1 year of CPAP treatment, the PSWEs decreased significantly. The results suggest that the PSWEs may be a marker for cognitive impairment in patients with OSA.

## Data Availability

The datasets used during the current study are available from the corresponding author on reasonable request.

## Abbreviations

Aβ: Amyloid-β
AASM: American Academy of Sleep Medicine
AD: Alzheimer’s disease
AHI: Apnea-hypopnea index
BBB: Blood-brain barrier
BMI: Body mass index
CPAP: Continuous positive airway pressure
CSVD: Cerebral small vessel disease
CyPA: Cyclophilin A
EEG: Electroencephalography
ESS: Epworth sleepiness scale
FLAIR: Fluid-attenuated inversion recovery
HAMA: Hamilton Anxiety Scale
HAMD: Hamilton Depression Scale
LDL-C: Low-density lipoprotein cholesterol
MCI: Mild cognitive impairment
mean SaO_2_: Mean oxygen saturation during sleep
min SaO_2_: Minimum oxygen saturation during sleep
MMP-9: Matrix metalloproteinase-9
MMSE: Mini-Mental State Examination
MoCA: Montreal Cognitive Assessment
MRI: Magnetic resonance imaging
NDE: Neuron-derived exosome
NREM: Nonrapid eye movement
ODI: Oxygenation desaturation index
OSA: Obstructive sleep apnea
PLM: Periodic limb movement
PSG: Polysomnography
PSWE: Paroxysmal slow wave event
REM: Rapid eye movement
RBD: Rapid eye movement sleep behavior disorder
RSWA: REM sleep without atonia
SWI: Susceptibility-weighted imaging
T90: Percentage of sleep time spent with oxygen saturation < 90%
TST: Total sleep time
WMH: White matter hyperintensity
